# What the World Said

**Published:** 1879-08

**Authors:** 


In publishing Mr. Chas. C. Waite's "Bill of Fare for Diabetics," which we reproduce in this issue of the Journal of Health, the daily *World* spoke editorially as follows:

"We have been fortunate enough to secure from a gentleman whose name is at the service of any responsible inquirer, a full and accurate *menu* of the dietary used by him during many months while under medical care for a disease characteristic of our times, if not our country, and commonly esteemed to be to all intents and purposes incurable. We feel that we are performing a public service in laying this *menu* before our readers, and we invite to it both the attention and the criticism of competent men of science."

The following day the editorial columns of the *World* contained the following:

"In reply to several letters on the subject yesterday received, we beg to say that upon consulting the gentleman of this city to whom we are indebted for the results of his experience for patients suffering from diabetes, which was yesterday published in the *World*, we learn that the very best gluten bread and flour ever used by him, either in this country or in Europe, was procured from the Health Food Company in Fourth Avenue, in this city. The business card of this company will be found in our advertising columns, but as their gluten is expressly recommended by the gentleman to whom we refer (and whose name is at the service of any person interested in the subject) we think it proper to state this fact, which is an essential part of the hygienic programme drawn up by him and by him kindly communicated to us for publication."

